# Endoscopic retrieval of an accidentally ingested bur during a dental procedure: a case report

**DOI:** 10.1186/s13037-020-00273-3

**Published:** 2021-01-05

**Authors:** Keerthana Kunaparaju, Karthik Shetty, Vinod Jathanna, Kartik Nath, Roma M

**Affiliations:** 1 Department of Conservative Dentistry and Endodontics, Manipal College of Dental Sciences, Mangalore, Affiliated to Manipal Academy of Higher Education, Manipal, Karnataka, India; 2 Faculty of Dentistry, Department of Conservative Dentistry and Endodontics, Melaka Manipal Medical College, Manipal, India

**Keywords:** Foreign body, Dental bur, Rubber dam, Esophagogastroduodenoscopy (EGD)

## Abstract

**Background:**

Accidental ingestion of a dental bur during the dental procedure is a rare, but a potentially serious complication. Early recognition and foreign body retrieval is essential to prevent adverse patient outcomes.

**Case presentation:**

A 76-year old male patient, presented to the department with a chief complaint of sensitivity in his upper right back tooth due to attrition. After assessing the pulp status, root canal therapy was planned for the tooth. During the procedure, it was noticed that the dental bur slipped out of the hand piece and the patient had accidentally ingested it. The patient was conscious and had no trouble while breathing at the time of ingestion of the bur although he had mild cough which lasted for a short duration. The dental procedure was aborted immediately and the patient was taken to the hospital for emergency care. The presence and location of the dental bur was confirmed using chest and abdominal x-rays and it was subsequently retrieved by esophagogastroduodenoscopy (EGD) procedure under general anaesthesia on the same day as a part of the emergency procedure. The analysis of this case reaffirms the importance of the use of physical barriers such as rubber dams and gauze screens as precautionary measures to prevent such incidents from occurring.

**Conclusion:**

Ingestion of instruments are uncertain and hazardous complications to encounter during a dental procedure. The need for physical barrier like rubber dam is mandatory for all dental procedures. However, the dentist should be well trained to handle such medical emergencies and reassure the patient by taking them into confidence. Each incident encountered should be thoroughly documented to supply adequate guidance for treatment aspects. This would fulfil the professional responsibilities of the dentist/ clinician and may help avoid possible legal and ethical issues. This case report emphasizes on the need for the usage of physical barriers during dental procedures in order to avoid medical emergencies.

## Background

Accidental ingestion of dental instruments by the patients during the procedure, though not common has been documented in literature over the years. All types and sizes of dental instruments such as orthodontic appliances [1], BiTine rings [2], dental burs [3], endodontic files [4, 5], rubber dam clamp [6], barbed broaches [7, 8], dental mirror [9], implant instruments [10], etc. have been shown to be ingested in reports. Suitable precautions to avoid foreign body ingestion should be practiced as a matter of standard practice. However, even under the most ideal circumstances, the possibility of accidentally dropping an instrument into the oral cavity is always a scenario which the clinician may have to face during his or her practice. In this case report we aim to describe the ingestion of a dental polishing bur by an elderly patient during post endodontic restoration procedure. A dental bur is a rotary instrument used for cutting, finishing and polishing the tooth and the restorations. A polishing bur is often used as the last rotary instrument to finish the final restoration.

## Case presentation

**Fig. 1:**
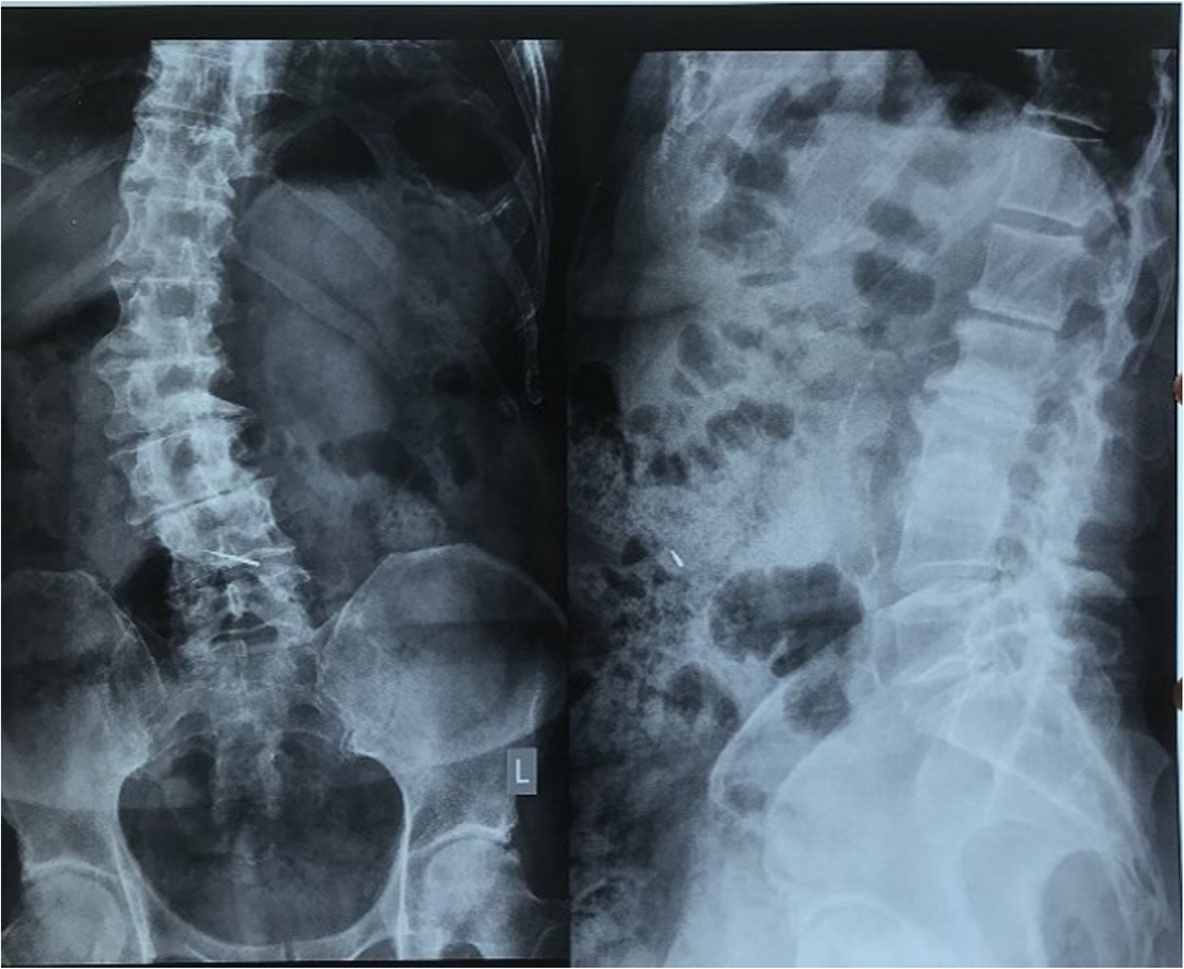
Abdominal X-ray image showing the presence of a linear pointed radiopaque foreign body in the anterior aspect of mid abdomen (L4 level)

A 76- year old male patient came to the dental clinic with a chief complaint of sensitivity in his upper right back tooth for last 1 year due to severe attrition. Intentional root canal therapy was planned for the maxillary right first molar tooth followed by a crown. Isolation with rubber dam was not possible as the tooth was tilted with considerable amount of tooth loss and missing adjacent tooth. During the post endodontic restoration, following the root canal treatment, the patient suddenly felt the presence of a foreign body in his throat and coughed momentarily attempting to spit it out. On examination the bur was found missing on the Airotor and was not detected in the oral cavity. The treatment was immediately stopped, patient was informed about the suspected bur drop. Following a chest and abdominal X-ray accidental ingestion of dental polishing bur [NMD high speed composite polishing and finishing kit (yellow band, diamond)] was confirmed. The abdominal X-ray showed the presence of linear pointed radiopaque foreign body in the anterior aspect of mid abdomen (L4 level) (Fig. 1).

**Fig. 2:**
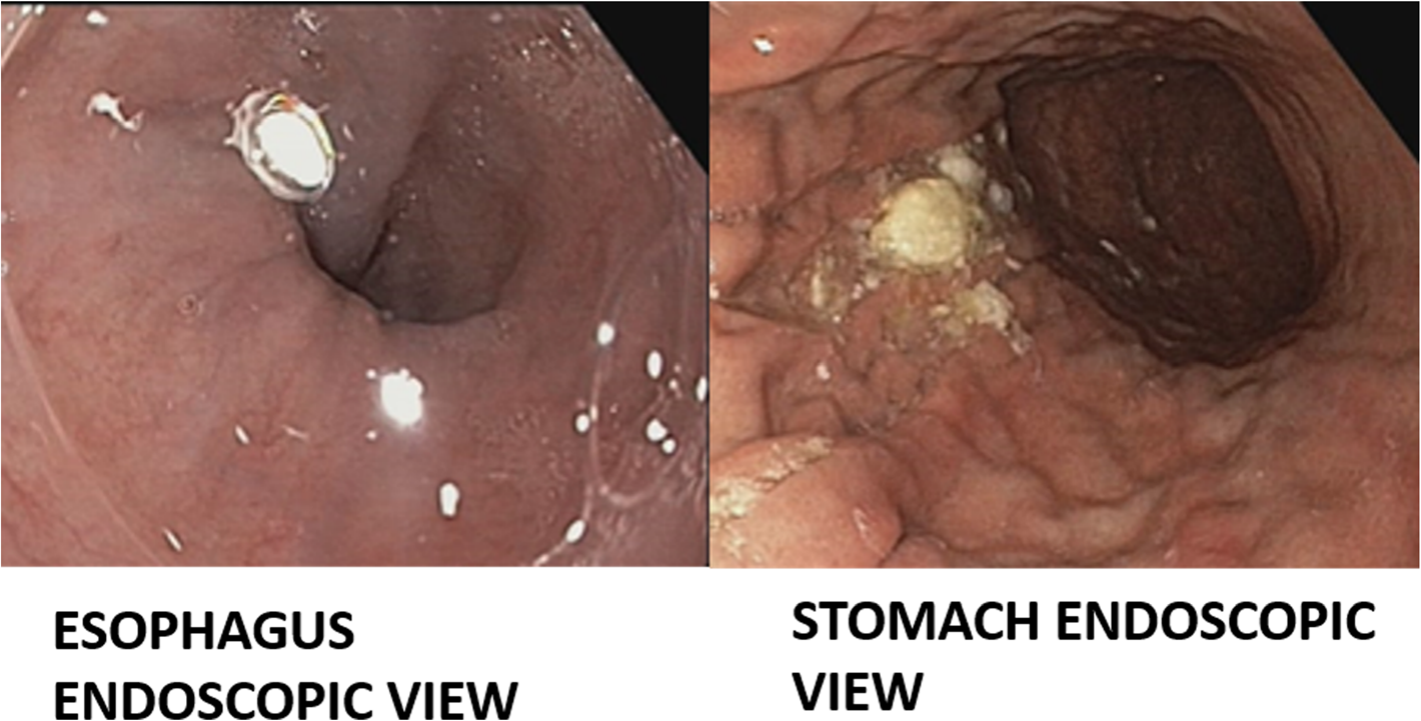
Gastroduodenoscopy image showing normal view of oesophagus, fundus, body and antrum of the stomach with no perforation

**Fig. 3:**
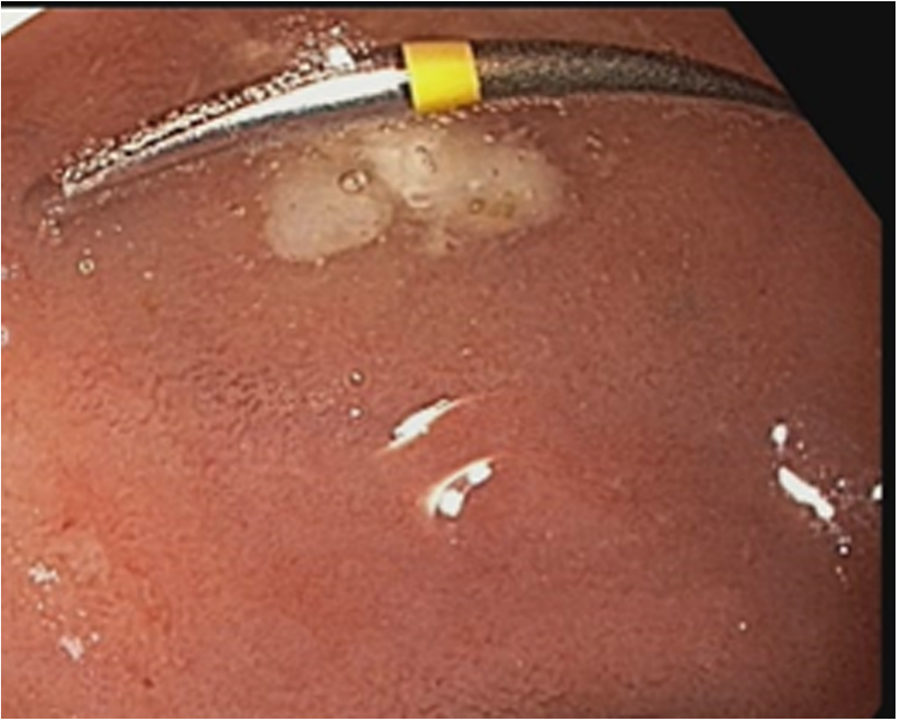
Gastroduodenoscopy image showing the presence of ingested dental bur in the duodenal bulb

**Fig. 4:**
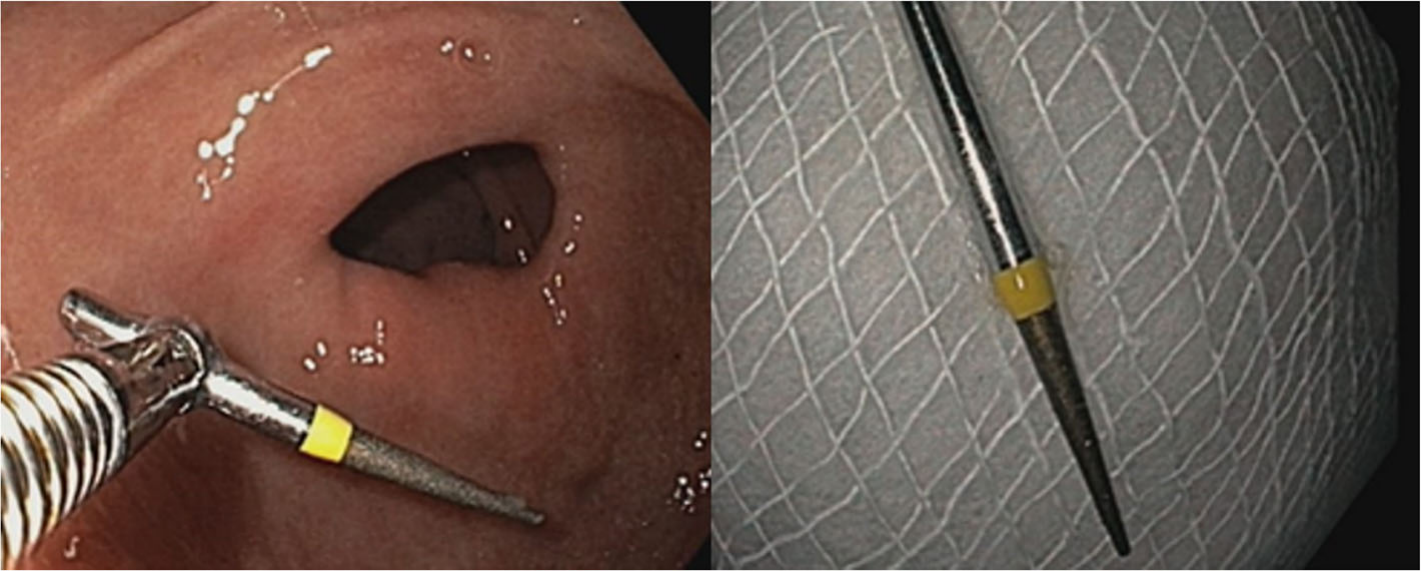
Gastroduodenoscopy image showing the presence of retrieved instrument using the rat tooth forceps

Since the patient had a history of bypass surgery and is hypertensive as well as diabetic, a complete blood picture and a cardiac echo were advised before proceeding with any procedure. After obtaining cardiac clearance and normal complete blood picture reports, esophagogastroduodenoscopy was planned under anaesthesia to remove the bur. The esophagogastroduodenoscopy report revealed that the oesophagus, fundus, body and antrum of the stomach were normal (Fig. 2). Perforation was not present. The foreign body was present in the duodenal bulb (Fig. 3). The endoscopy was performed under general anaesthesia and the foreign body was extracted using the rat tooth forceps (Fig. 4). After the endoscopy patient was discharged without complications and was kept under follow up. Upon 3 months follow up period, the patient was asymptomatic.

## Discussion

Accidental aspiration or ingestion of foreign bodies is a potential event encountered across all age groups. It may affect geriatric and pediatric patients, mentally challenged or physically disabled people whose coordination or control of deglutition is impaired [11–13]. Rui et al. evaluated the different variables associated with the instrument aspiration and ingestion. According to them the age groups of 60–79 years and 10–19 years, showed high incidence of aspirations and ingestions [13]. In older patients, the risk is higher due to the reduced gag reflex and other age-related general diseases, such as dementia or Parkinson’s disease [14]. One other possibility in this case is that the local anaesthesia used for the dental procedure might also have compromised the protective gag reflex [3]. It has been reported that the majority of small sized foreign objects of less than 2 mm in thickness can easily pass out through the alimentary canal [15]. If the foreign instrument gets lodged into deeper tissues, it can result in complications like intestinal perforations, stricture formation, abdominal pain, etc. for which surgical intervention may be required [16]. Some of the operative procedures have a greater risk of foreign body aspiration, it is important to inform the patient, and their relatives, about the possible risks pertaining to the procedure. Informed consent should be taken from the patient in both verbal and written format [17]. Whenever there is accidental slippage of instrument during the procedure in the patient’s mouth one should take care to tilt the patient’s head to the sideways, so that the foreign object dropped is collected near the side of the mouth and does not enter the oropharynx. When the accidental mishap happens, the concerned professional should observe the response of the patient by communicating and check for breathing pattern. In case of emergency situation, the professional is bound to perform Heimlich manoeuvre to remove the object, and must contact for immediate assistance [18]. This case reports highlights the rare clinical scenario of accidental ingestion of dental bur and the fundamental concepts need to be followed while treating the patient for early intervention of ingested instruments.

## Conclusion

This case report further highlights the importance of additional caution and usage of some form of protective physical barrier such as a curtain of gauze, especially in geriatric patients, when a rubber dam placement may not be easily possible to use. A thorough check-up of the handpiece and other instruments must be done before every use in order to avoid operator negligence. This case report also emphasizes on the importance of gastroduodenoscopy as an interceding diagnostic tool as well as an intervening modality for the retrieval of ingested objects.

## Data Availability

Data sharing is not applicable to this article as no datasets were generated or analysed during the current study.

## References

[1] Rohida NS, Bhad WA (2011). Accidental ingestion of a fractured twin-block appliance. Am J Orthod Dentofac Orthop.

[2] Baghele O, Mangala OB (2011). Accidental ingestion of BiTine ring and a note on inefficient ring separation forceps. Ther Clin Risk Manag.

[3] Sankar NS (1998). Accidental ingestion of a dental instrument. J R Soc Med.

[4] Kuo SC, Chen YL (2008). Accidental swallowing of an endodontic file. Int Endod J.

[5] Saraf HP, Nikhade PP, Chandak MG. Accidental ingestion of endodontic file: a case report. Case Rep Dent. 2012:1–3. 10.1155/2012/278134. 10.1155/2012/278134 PMC3337583 22577586

[6] Mejia JL, Donado JE, Posada A (1996). Accidental swallowing of a dental clamp. J Endod.

[7] Govila CP (1979). Accidental swallowing of an endodontic instrument. A report of two cases. Oral Surg, Oral Med Oral Pathol.

[8] Mohan R, Benjamin M, Rao S, Bhagavan R (2011). Accidental ingestion of a barbed wire broach and its endoscopic retrieval: prevention better than cure. Indian J Dent Res.

[9] Oncel M, Apiliogullari B, Cobankara FK, Apiliogullari S (2012). Accidental swallowing of the head of a dental mirror: report of a rare case. J Dent Sci.

[10] ter Gunne LP, Wismeijer D (2014). Accidental ingestion of an untethered instrument during implant surgery. Int J Prosthodont.

[11] Mahesh R, Prasad V, Menon PA (2013). A case of accidental aspiration of an endodontic instrument by a child treated under conscious sedation. Eur J Dent.

[12] Deliberador TM, Marengo G, Scaratti R, Giovanini AF, Zielak JC, Baratto FF (2011). Accidental aspiration in a patient with Parkinson's disease during implant-supported prosthesis construction: a case report. Spec Care Dentist.

[13] Hou R, Zhou H, Hu K, Ding Y, Yang X, Xu G, Xue P, Shan C, Jia S, Ma Y (2016). Thorough documentation of the accidental aspiration and ingestion of foreign objects during dental procedure is necessary: review and analysis of 617 cases. Head Face Med.

[14] Fields RT, Schow SR (1998). Aspiration and ingestion of foreign bodies in oral and maxillofacial surgery: a review of the literature and report of five cases. J Oral Maxillofac Surg.

[15] Birk M, Bauerfeind P, Deprez PH, Häfner M, Hartmann D, Hassan C, Hucl T, Lesur G, Aabakken L, Meining A (2016). Removal of foreign bodies in the upper gastrointestinal tract in adults: European Society of Gastrointestinal Endoscopy (ESGE) clinical guideline. Endoscopy.

[16] Weiland ST, Schurr MJ (2002). Conservative management of ingested foreign bodies. J Gastrointest Surg.

[17] Silva RF, Martins EC, Prado FB, Júnior JR, Júnior ED (2011). Endoscopic removal of an endodontic file accidentally swall­owed: clinical and legal approaches. Aust Endod J.

[18] Cameron SM, Whitlock WL, Tabor MS (1996). Foreign body aspiration in dentistry: a review. J Am Dent Assoc.

